# Droplet Digital PCR for the Detection of *Plasmodium falciparum* DNA in Whole Blood and Serum: A Comparative Analysis with Other Molecular Methods

**DOI:** 10.3390/pathogens9060478

**Published:** 2020-06-17

**Authors:** Elena Pomari, Ronaldo Silva, Lucia Moro, Giulia La Marca, Francesca Perandin, Federica Verra, Zeno Bisoffi, Chiara Piubelli

**Affiliations:** 1 Department of Infectious, Tropical Diseases and Microbiology, IRCCS Sacro Cuore Don Calabria Hospital, Negrar di Valpolicella, 37024 Verona, Italy; ronaldo.silva@sacrocuore.it (R.S.); lucia.moro@sacrocuore.it (L.M.); giulia.lamarca@sacrocuore.it (G.L.M.); francesca.perandin@sacrocuore.it (F.P.); fverra66@gmail.com (F.V.); zeno.bisoffi@sacrocuore.it (Z.B.); 2 Department of Diagnostics and Public Health, University of Verona, 37134 Verona, Italy

**Keywords:** *Plasmodium falciparum*, DNA, absolute quantitation, droplet digital PCR, microscopy

## Abstract

Background: The estimation of *Plasmodium falciparum* parasitaemia can vary according to the method used. Recently, droplet digital PCR (ddPCR) has been proposed as a promising approach in the molecular quantitation of *Plasmodium*, but its ability to predict the actual parasitaemia on clinical samples has not been largely investigated. Moreover, the possibility of applying the ddPCR-sensitive method to serum samples has never been explored. Methods: We used, for the first time, ddPCR on both blood and serum to detect the DNA of *P. falciparum* in 52 paired samples from 26 patients. ddPCR was compared with loop-mediated isothermal amplification (LAMP) and rtPCR. The correlation between the ddPCR results, microscopy, and clinical parameters was examined. Results: ddPCR and microscopy were found to be strongly correlated (ρ(26) = 0.83111, *p* < 0.0001) in blood. Samples deviating from the correlation were partially explained by clinical parameters. In serum samples, ddPCR revealed the best performance in detecting *P. falciparum* DNA, with 77% positive samples among malaria subjects. Conclusion: Absolute quantitation by ddPCR can be a flexible technique for *Plasmodium* detection, with potential application in the diagnosis of malaria. In particular, ddPCR is a powerful approach for *Plasmodium* DNA analysis on serum when blood samples are unavailable.

## 1. Introduction

Malaria is a major human infectious disease, it is caused by intracellular protozoan parasites of the genus *Plasmodium* and it is transmitted by *Anopheles* mosquito species. There are five parasite species that cause malaria in humans, and one of these species—*P. falciparum*—poses the greatest threat [1,2]. According to World Health Organization (WHO) in 2017, 219 million malaria cases have been estimated, causing 435,000 deaths worldwide [3]. The incidence of malaria globally decreased between 2010 and 2017, but in recent years this trend has slowed dramatically. To accelerate progression towards malaria eradication, multiple interventions are needed, but an accurate and sensitive surveillance of malaria transmission is essential to guide them [4]. On the other hand, Europe and other non-endemic countries nowadays face renovated malaria challenges; the increased influx of refugees, migrant populations, and travelers from endemic areas can contribute to the build-up of an infectious parasite reservoir, and climate change might favor mosquitos’ proliferation and parasite development, further facilitating malaria transmission [5,6,7,8,9,10]. A prompt and accurate diagnosis of malaria is part of effective disease management [4,11]. The diagnosis of malaria is based on clinical criteria (clinical diagnosis) and traditional microscopy for the detection (thick blood smear) and identification (thin blood smear) of *Plasmodium* species. However, microscopy’s main shortcoming is its low sensitivity, especially at low parasite density [12,13]. The severity of clinical symptoms and transmission potential are both closely related to parasite densities. Parasite densities also show pronounced age patterns, reflecting lifetime exposure and naturally acquired “premunition” at a population level. The density of malaria parasites in the blood of infected humans ranges from below 1 parasite/μL to tens of thousands of parasites/μL [14]. Alternative techniques for the laboratory diagnosis of malaria are rapid diagnostic tests (RDTs) for antigen detection, but they do not necessarily offer improved sensitivity over microscopy, which has a limit of detection of ~50 parasites/μL of blood. On the contrary, polymerase chain reaction (PCR) has demonstrated a high sensitivity and specificity for the detection of *Plasmodium* and is traditionally recommended for epidemiological research and surveys of sub-microscopic infections [15,16]. The most widely used molecular assay is quantitative real-time PCR (rtPCR), which has been used for species identification and relative quantitation. Target genes include *18S rRNA* (the most frequently used), *tRNA*, *cytochrome b*, *ama1,* and *stevor* [17]. On the basis of recent advances in molecular biology and keeping in mind the limitations of conventional methods, additional techniques have been introduced for the DNA-based detection of *Plasmodium,* such as loop-mediated isothermal amplification (LAMP) and droplet digital PCR (ddPCR) [18]. Studies have shown the efficacy of all these molecular techniques in detecting *Plasmodium* DNA extracted from whole blood. The following limits of detection have been reported: 0.2 parasites/μL of blood for LAMP [19], 0.02 parasites/μL for rtPCR [20,21], and 0.01 parasites/μL for ddPCR [17], where the latter two values have been obtained in concentrated erythrocyte samples. Although *P. falciparum* DNA has been mostly explored in whole blood, some studies in the literature reported that it may be also detected in serum and plasma using conventional PCR and rtPCR [22,23,24,25]. This nucleic acid presumably derives from degraded intraerythrocytic parasites and free merozoites, and some investigations have suggested that its concentration may be a better measure of the disease severity [25]. The expected plasmodium DNA concentration in serum is not high, so a very sensitive molecular method should be used for quantifying it. Thus, we decided to evaluate the ddPCR technology for the detection of *P. falciparum* DNA in serum specimens from malaria patients. The corresponding whole blood samples were also analyzed. The ddPCR data were compared with other two molecular methods (LAMP and rtPCR) on the same samples. Moreover, the ddPCR results were related to microscopy and clinical data in order to evaluate its capability of assessing the actual parasitaemia and possibly the disease severity or clinical complications.

## 2. Results

### 2.1. Clinical Description

Twenty-six (P1–P26) malaria patients were enrolled in the study, and all were *P. falciparum*-positive based on microscopy. Their clinical, demographical, and diagnostic data are reported in Table 1 and in Supplementary Materials Table S1. Ten patients were Italian and 16 had an African origin. All were exposed to *P. falciparum* malaria in the African continent. Nine subjects had a parasitaemia of <0.1% (17–4620 parasites/μL of blood). All the patients received standard care and responded to the treatment. Eight complicated cases required therapy with intravenous artesunate for different reasons, of which three met the WHO definitions of severe malaria (one for impaired consciousness, one for multiple convulsions, and one for a parasitaemia of above 10% of infected Red Blood Cells, iRBCs). Five of the total (*n* = 26) had taken chemoprophylaxis, but with inadequate compliance (four with mefloquine and one with doxycycline). Moreover, seven additional patients (P27–P33) of African origin were enrolled for the predictive analysis, and they were *P. falciparum*-positive based on microscopy. Their clinical and demographical descriptions are reported in Table S1.

### 2.2. Limit of Detection Analysis

In order to assess the limit of detection, we first tested the ddPCR as well as LAMP and rtPCR on *P. falciparum*-infected blood in 10-fold serial dilutions (100 to 0.01 parasites/μL density). The ddPCR and rtPCR were tested in six replicates per concentration, and both showed a good linearity with a reproducible signal in all the six replicates until 0.1 parasites/μL of blood (Figure S1 and Table S2). LAMP was used in three replicates of measurement and was able to detect a signal until 0.1 parasites/μL of blood (data not shown).

### 2.3. Detection of P. falciparum DNA in Whole Blood

**Figure 1:**
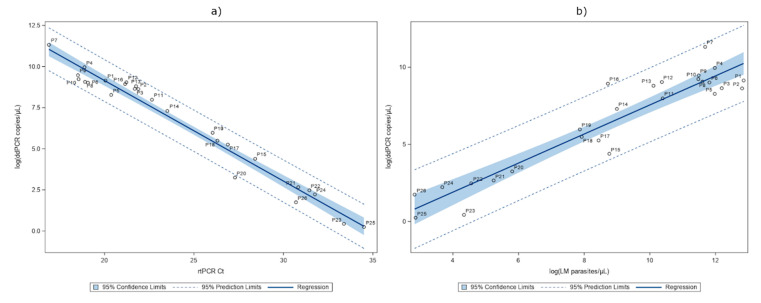
Quantitation of parasites by ddPCR vs. real-time PCR (rtPCR) (**a**) on blood samples (parameter estimate: N = 26, intercept 21.37; rtPCR Ct −0.611, R^2^ = 0.9682). The quantitation of parasites by ddPCR vs. light microscopy (LM) (**b**) (parameter estimate: N = 26, intercept −1.87; logLM parasites/μL 0.942, R^2^ = 0.8844). LM data was reported as Log(parasites/μL), the rtPCR was reported as the Ct value, and the ddPCR data were reported as Log(DNA copies/μL).

We analyzed whole blood samples from 26 patients (P1–P26) with all the three molecular methods, and the results are summarized in Table 2. ddPCR as well as LAMP and rtPCR detected a *Plasmodium*-positive signal in all the whole blood samples analyzed, according to the microscopy. For ddPCR, three samples (P4, P7, P16) were diluted to avoid *P. falciparum* signal saturation. The ddPCR and rtPCR were found to be strongly correlated, ρ(26) = −0.97675, *p* < 0.0001). The ddPCR provided signals ranging from 82,500 to 1.276 gene copies/μL of blood and the rtPCR from 16.89 to 34.50 Ct values. The numerical association between these two tests are summarized with a linear regression of Ct-values against the log copies/μL of blood, giving an R^2^ of 0.9682 (Figure 1a). The ddPCR and microscopy counting (as parasites/μL of blood) were also found to be strongly correlated (ρ(26) = 0.83111, *p <* 0.0001). The numerical associations between these two tests in log scale are summarized in Figure 1b, with a linear regression giving an R^2^ of 0.8844. Two samples (P7, P16) showed a higher DNA detection by ddPCR than expected compared to microscopy counting. On the other hand, the other two cases (P15 and P23) presented a lower signal with respect to the detected parasitaemia by microscopy.

The LAMP reactions were considered positive or negative for the detected presence or absence of Plasmodium DNA respectively. The rtPCR results were negative if no amplification was detected or if the Ct ≥ 46 cycles. The ddPCR results were calculated based on the proportion of positive or negative droplets. A ddPCR reaction was considered positive if at least 1 copy/μL was quantified.

### 2.4. Detection of P. falciparum DNA in Serum

**Figure 2:**
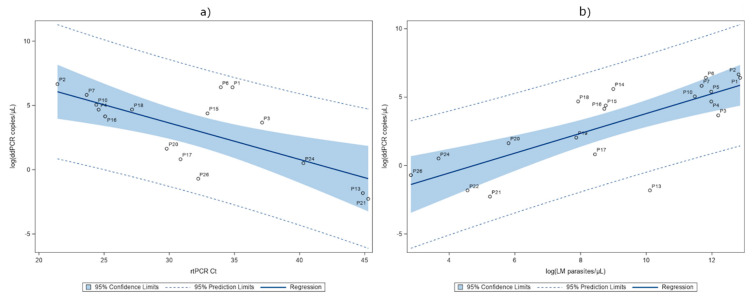
Quantitation of parasites by ddPCR vs. real-time PCR (rtPCR) (**a**) on serum samples (parameter estimate: N = 16, intercept 12.15; rtPCR Ct −0.284, R^2^ = 0.4871). Quantitation of parasites by ddPCR vs. light microscopy (LM) (**b**) on serum samples (parameter estimate: N = 20, intercept −3.44; logLM parasites/μL 0.724, R^2^ = 0.5875). LM data was reported as Log(parasites/μL), the rtPCR was reported as the Ct value, and the ddPCR data were reported as Log(DNA copies/μL).

**Figure 3:**
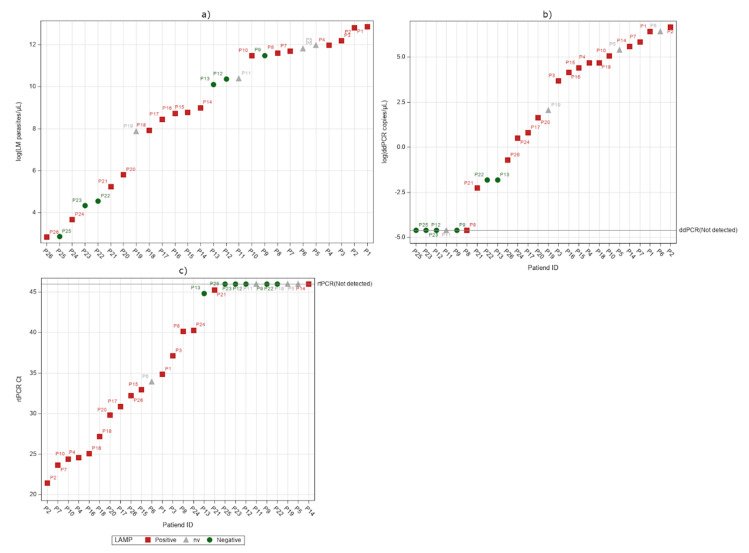
Detection of *Plasmodium* DNA by LAMP in serum. The results are plotted against the microscopy Log(parasites/μL of blood) (**a**), rtPCR (Ct value on serum) (**b**), and ddPCR Log(copies/μL of serum) (**c**). An arbitrary offset was added in order to plot the ddPCR and rtPCR not-detected results.

We analyzed serum samples from the same 26 patients (P1–P26) with all the three molecular methods and the results are summarized in Table 2. The ddPCR results were found to be strongly correlated to those obtained with rtPCR (ρ(16) = −0.60882, *p* = 0.0123). The ddPCR and rtPCR detected 77% (20/26) and 65% (17/26) of the positive samples of the microscopy, respectively. The numerical association between these two tests in log scale are summarized with a linear regression, giving an R^2^ of 0.4871 (Figure 2a). The ddPCR and microscopy counts were also strongly correlated (ρ(20) = 0.75141, *p <* 0.0001). The numerical association between these two tests in log scale are summarized in Figure 2b, with a linear regression giving an R^2^ of 0.5875. Six (P8, P9, P11, P12, P23, P25) out of 26 serum samples were not detected by ddPCR, independently from the microscopy parasitaemia range. Using the LAMP, 62% (16/26) of the samples were positive and 15% (4/26) provided “non-valid” results (Figure 3a). Figure 3a,c shows the relationship between LAMP and the other two PCR methods. 

## 3. Discussion

We conducted the first study to investigate ddPCR technology in the detection of *P. falciparum* DNA in the whole blood and serum-paired samples of symptomatic patients, with the microscopy-confirmed parasitaemia ranging from 17 to 383,460 parasites/μL of blood. LAMP and rtPCR were also performed on the same samples. We first characterized the blood samples and, as expected for the considered range of parasitaemia, LAMP was as sensitive as the other PCR techniques using blood specimens. We observed an equal LOD using all the three molecular methods, suggesting that a supplementary concentration of erythrocytes or DNA could be useful, as reported in literature with both rtPCR [20,21] and ddPCR [17], to improve the sensitivity in blood samples. Of note, LAMP has the distinct advantage of working at a single temperature, but it does not allow either the species identification or the quantitative screening. In contrast, the other two techniques produced semi-quantitative/quantitative data, and the linear regression analysis showed a good numerical association between rtPCR and ddPCR in revealing *P. falciparum* DNA (Figure 1a). Then, comparing the quantitative molecular data of ddPCR [as log(copies/μL of blood)] with the microscopy count [as log(parasites/μL of blood)], we observed a significant correlation (Figure 1b). We tested our model’s ability to predict the microscopy count for a given quantity of copies/μL of blood. We used additional whole blood samples collected from seven patients (P27–P33) (Table S3) and a dataset reported in a previously published study [26]. The results (Figure S2) showed that the majority (27 out of 35) of the predicted values fall into the limit of the 95% confidence interval of the observed values. Since the dataset is still limited, we created a dedicated website in which also external users could add their own digital PCR data on malaria samples and elaborate data with our statistical model (https://ditm.shinyapps.io/ditm/). Populating the dataset will allow a better evaluation of the ddPCR tool for calculating parasitaemia or highlighting discrepancies with microscopy. In fact, also in our data set some cases resulted in being discordant with respect to the expected range of parasitaemia measured by microscopy. An interesting example is represented by P7. We found an unexpected very high molecular signal (the highest of the whole set of samples) from both the rtPCR and ddPCR, despite a parasitaemia of 3.4% (118,800 parasites/μL of blood). After retrieving the clinical description, we realized that the sample was from a pregnant woman, and it is known that microscopic counting can underestimate the parasitaemia in infected pregnant women due to a retention of parasites in the placenta [27]. In another case (P16), the presence of schizonts (confirmed by microscopy) [28,29,30,31] can explain the high molecular signal detected by ddPCR. On the contrary, in two Italian subjects (P15 and P23) who had multiple episodes, followed chemoprophylaxis, and were without complications, the ddPCR signal was lower than expected, thus indicating a possible lower yield in the DNA extraction for these samples. Overall, the discordant results with a higher ddPCR signal could be explained by a lower precision of microscopy counting in high parasitaemia samples or by the fact that the molecular method can detect the presence of any circulating form of *Plasmodium* DNA (also from degraded parasites) [13,28]. Moreover, it is known that microscopy can underestimate parasitaemia in some disease stages when infected erythrocytes are sequestered within different body organs [32]. In the case of patients with very low parasitaemia, a method that can improve the sensitivity of the molecular tests is desirable. Recent evidence has demonstrated that ddPCR may be especially useful for the screening of low-level parasitaemia due to its analytical sensitivity [18] compared especially to quantitative rtPCR. It has been shown that quantitative rtPCR is more sensitive than thick-film microscopy in whole blood and has a good agreement also with plasma samples [24]. In our study, a serum analysis revealed that ddPCR could detect a higher number of positive samples than rtPCR and LAMP, including 88% (7/8) of the complicated cases (serum samples P1–P5, P7, P13). For the sake of clarity, we used a LAMP kit validated only for whole blood and modified the protocol to adapt it to serum, adding genomic DNA as an internal control. This could be the potential reason for the presence of four non-valid LAMP results in serum samples. By ddPCR, we could detect *Plasmodium* DNA in 20 out of 26 malaria serum samples (77% of cases). By a linear regression analysis, we found that the quantitative results of the ddPCR assay in serum had a moderate but significant correlation with the microscopy counting (*p* < 0.0001, Figure 2b). As expected, *P. falciparum* DNA was not detected in all of the serum samples; in fact, in 23% it was not detected, thus confirming that serum is not an ideal biological specimen when high sensitivity is needed. Moreover, from our analysis no correlation was found between the ddPCR values in serum samples and either the disease severity or any other measured parameter, such as bilirubin or lactate dehydrogenase (LDH) (analysis not shown). This notwithstanding, our results support previous evidence [22] suggesting that *P. falciparum* DNA can be explored in archival stored samples to be used, for instance, in retrospective epidemiological and phylogenetic studies of the parasite.

## 4. Materials and Methods

### 4.1. Setting and Participants

We analyzed a total of 33 symptomatic patients diagnosed with *P. falciparum* malaria at our Department of Infectious Tropical Diseases and Microbiology, located in Negrar, northern Italy, from January 2014 to September 2018. All the patients were exposed in the African continent. Specifically, we divided them in two sets of patients: set 1 of 26 (P1–P26) (Table 1 and Table S1) individuals for the ddPCR analysis on whole blood and serum, and the same samples were analyzed also by LAMP and rtPCR as reference molecular methods; and set 2 of an additional 7 individuals (P27–P33) (Table S1), who were included to test the ability of our model to predict microscopy values from the ddPCR analysis on blood. As a routine laboratory approach, for each patient 3 mL of fresh blood in EDTA was collected and examined using light microscopy. At the same sampling, serum was also collected. The blood and serum samples were de-identified, divided immediately in aliquots and stored at −80 °C until the DNA extraction/molecular analyses. The data were archived in an electronic database. This study was approved by the local competent Ethics Committee for Clinical Research of Verona and Rovigo Provinces (protocol no.43352, 2018). The blood and serum samples were collected in accordance with the requirements of the Declaration of Helsinki; all the adult patients included in this study gave their written consent for research purposes. Four children were also included in the study, and for them the written informed consent of parents/legal representatives was obtained on their behalf.

### 4.2. Diagnostic Analysis

The laboratory screening of all the patients included the following diagnostic analyses. The determination of antibodies to *P. falciparum* in the fresh serum of patients was performed using an enzyme immunoassay with the Malaria Ab kit (DIAPRO) according to the manufacturers’ instructions. The OD results were normalized versus the cut-off control sample. Samples with normalized values ≥1 were considered positive. The haemolysis was determined by the measurement of bilirubin (healthy range 5–21 μmol/L) and lactate dehydrogenase (LDH) (healthy range for adults 140–250 U/L, for children ≤360 U/L). All the patients were also diagnosed by clinical observation.

### 4.3. Analysis of Samples by Microscopy

The thick blood films from blood samples were allowed to dry, then stained with 2.5% Giemsa for 15 min. The thin blood films were fixed with methanol and stained with 10% Giemsa for 25 min. The parasitaemia was later determined by experienced microscopists. The parasitaemia was calculated by counting up to 200 white blood cells (WBCs), the corresponding number of malaria parasites in the thick blood films, and by using each patient’s actual WBC count for calculating the number of trophozoites per μL of blood. The percent of iRBCs was determined by calculating the number of infected RBCs in relation to the total number of RBCs.

### 4.4. Analysis of Samples by RDT

The antigen-based RDT BinaxNOW^®^ Malaria (Abbott) was used according to the manufacturers’ instructions. The fresh blood samples from malaria patients were processed within three hours of collection. BinaxNOW^®^ Malaria requires 15 μL of blood and takes 15 min to complete. Tests were interpreted as successful when the control band was positive.

### 4.5. LAMP

The whole blood (50 μL) and serum (50 μL) specimens were processed using the commercial Illumigene Plus Malaria LAMP kit (Meridian Bioscience, Milan, Italy), following the manufacturer’s instruction. This assay targets a 214  base pair sequence of a *Plasmodium* sp. mitochondrial DNA noncoding region that is conserved across *P. falciparum*, *P. vivax*, *P. ovale*, *P. malariae,* and *P. knowlesi*. The exact sequences of the primers are not available from Meridian Inc. as part of intellectual property rights. All the analyses were performed using the illumipro-10™. Incubator/Reader with a temperature of 63 °C. Each run contained a negative control with no *P. falciparum* DNA (from malaria-naïve donor) and a positive control with *P. falciparum* DNA. Only for the analyses on serum, 1 μg of genomic DNA (isolated from whole blood of malaria-naïve donors) was added to each specimen. The LAMP reactions were considered positive or negative for the detected presence or absence of *Plasmodium* DNA, respectively. For the limit of detection analysis, the LAMP assay was tested on *P. falciparum*-infected blood in 10-fold serial dilutions (100 to 0.001 parasites/μL of blood). Each concentration was tested by three replicates. The “non-valid” results were repeated.

### 4.6. DNA Extraction

For DNA extraction, 200 μL of whole blood and 500 μL of serum were used. After thawing, the samples were transferred to the cartridge sample of a MagnaPureLC.2 instrument (Roche Diagnostic, Monza, Italy). For whole blood samples, this was followed the protocol DNA_I_Blood_Cells_High performance_II, using the DNA isolation kit I (Roche) with a final elution volume of 100 μL; for serum samples, we was followed the protocol DNA_ Blood_300–500, using the DNA isolation kit LV (Roche) with a final elution volume of 200 μL. The DNA samples were stored at −20 °C until further PCR analysis.

### 4.7. Real-Time PCR

The rtPCR assay was performed as described by Perandin et al. [33]. The small-subunit (SSU) rRNA gene sequences was chosen as the amplification target. The primers and probes were synthesized by Eurofins Genomics (Ebersberg, Germany). Amplification reaction was performed in a volume of 50 μL using 5 μL of DNA from blood or 20 μL of DNA from serum; the PCR cycle protocol consists of 3 min at 95 °C followed by 46 cycles of 15 s at 95 °C and 1 min at 60 °C. The reactions, detection, and data analysis was performed with the CFX96 detection system (Bio-Rad Laboratories, Milan, Italy) using the white plates. As a control for PCR inhibitors and amplification quality, the β-actin gene [34] rtPCR was performed with the appropriate primers/probe mix, already used and validated in our routine screening, in a separate reaction PCR mix. The primers and probes are reported in Table S4. Each run contained a negative control with no template DNA, a negative control with no *P. falciparum* DNA (from malaria-naïve donor), and a positive control with *P. falciparum* DNA. The PCR results were stratified into high (Ct < 30), moderate (30 ≤ Ct ≤ 35), and low (Ct > 35) DNA load and negative (no amplification detected or Ct ≥ 46 cycles). For the limit of detection analysis, the rtPCR assay was tested on DNA extracted from *P. falciparum* infected blood in 10 fold serial dilutions (100 to 0.001 parasites/μL density). Each concentration was tested by six replicates.

### 4.8. ddPCR

The ddPCR for *P. falciparum* was conducted using a set of primers and a specific probe targeting the 18S ribosomal RNA gene of *P. falciparum* [33] with Bio-Rad Supermix for Probes (No dUTP) and primers/probe as used for rtPCR as described above and in Table S4. The AP3B1 gene was analyzed as an internal control gene using a specific set of primers/probe (Bio-Rad). The reactions, detection and data analysis was performed with the QX200 ddPCR (Bio-Rad). Each run contained a negative control with no template DNA, a negative control with no *P. falciparum* DNA (from malaria-naïve donor) and a positive control with *P. falciparum* DNA. In this assay, the ddPCR reaction was prepared in total 22 μL per reaction containing the ddPCR reagents (Bio-Rad 1X ddPCR Supermix, 0.9 μM of forward and reverse primers, and 0.25 μM of probe) and 5 μL of the DNA sample (<250 ng). Droplets were transferred to a PCR plate and standard PCR was performed using a C1000 thermal cycler (Bio-Rad). To optimize the PCR annealing temperature, a temperature gradient PCR of 60 °C, 58 °C, 56 °C, and 55 °C was used. An optimized PCR annealing temperature of the assay was 58 °C, which provided a clear separation between DNA positive and negative droplets. The conventional PCR was run with 95 °C for 10 min, 94 °C for 30 s, and 58 °C for 60 s in 40 cycles, and 98 °C for 10 min. After 40 cycles of PCR, DNA targets in each droplet were amplified and then analyzed by the Bio-Rad QX200TM Droplet Reader. The ddPCR results were calculated based on the proportion of positive or negative droplets. A ddPCR reaction was considered positive if at least three droplets were found positive. We converted the results of 18S rRNA concentration from ddPCR to 18S rRNA copies/μL of blood samples by using the formula: 18S rDNA copies/μL = [copies/μL from ddPCR × 22 (total volume of ddPCR in μL)]/[5 (Total loaded DNA in μL) × 2 (2× concentrated DNA)]. For the serum samples, the formula was: 18S rDNA copies/μL = [copies/μL from ddPCR × 22 (total volume of ddPCR in μL)]/[5 (Total loaded DNA in μL) × 5 (5× concentrated DNA)]. For the limit of detection analysis, the ddPCR assay was tested on DNA extracted from *P. falciparum*-infected blood in 10-fold serial dilutions (100 to 0.001 parasites/μL of blood). Each concentration was tested by six replicates.

### 4.9. Statistical Analysis

The demographic and clinical data are presented as absolute and relative frequency or median and first and third quartile (IQR). We used the SAS software v9.4 (SAS Institute, Inc., Cary, NC, USA) for the statistical analysis and plots. Spearman’s correlation and regression analysis (proc reg in SAS) were used to assess the numerical association between: ddPCR against rtPCR or microscopy counts on both blood and serum samples. ddPCR and microscopy data were log transformed. Standard statistical procedures were used for the model diagnostics (model assumptions, plots, *p*-value, and R^2^). We used a separated dataset to test one of our model’s ability (using the same model specification) to predict the microscopy count for a given quantity of copies/μL of blood. The prediction was obtained by setting the response variable as missing and using the output statement and the Y-predicted option of proc reg in SAS. Not detected ddPCR and rtPCR values on serum samples were excluded from regressions models. The statistical significance was fixed at 0.05.

## 5. Conclusions

Our analyses indicate that ddPCR is a sensitive method that can be applied for malaria diagnosis, in order to better evaluate the actual *Plasmodium* infection level, especially when the parasitic load in blood is low and the microscopy counting can underestimate the parasitaemia, such as, for instance, in cases in which parasites are sequestered by organs. To this purpose, ddPCR represents a powerful tool that can provide sensitive absolute quantitation data, with several potential applications, such as the identification of asymptomatic parasitaemic patients or the screening of blood donors at risk for transfusion-transmitted malaria. A limitation of our study is the restricted number of analyzed samples. A larger set of samples, including low-parasitaemic and malaria-naïve subjects, will allow a better evaluation of the sensitivity and specificity of the ddPCR assay. Further studies will be planned for this purpose. Moreover, we proposed a dedicated on-line tool for the collection of ddPCR data from different laboratories, in order to enlarge the sample set and to improve the ability to predict the real parasitaemia from molecular data. Regarding the serum analysis, we found that the 77% of the malaria samples returned a detectable *Plasmodium* DNA signal, thus indicating that ddPCR could be indeed applied in high-throughput format on this biological matrix, for example, for retrospective epidemiological studies in banked samples.

## Figures and Tables

**Table 1:** Characteristics of patients (*n* = 26) with *P. falciparum*-positive microscopy.

	Patients with *P. falciparum* Malaria N (%) or Median(IQR)
Patients	26 (100)
Country of origin 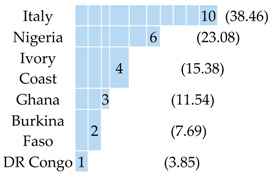	Country of exposition 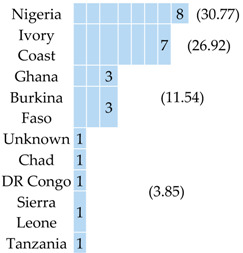
Sex M	20 (76.92)
Age by sex Male: Female:	49 (37–58) 37 (26–38)
Parasitaemia (trophozoites/μL of blood) at diagnosis	From 438,900 to 17
Microscopy identification *P. falciparum:*	26 (100)
*Plasmodium* antigen test result (BinaxNOW^®^ Malaria) 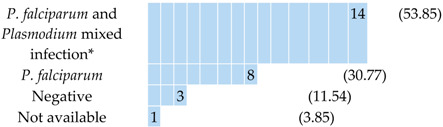	
LAMP (results on blood) *Plasmodium:*	26 (100)
Severe clinical manifestations	3 (11.54)
Pregnancy	1 (3.85)

Note: * mixed infections not confirmed by microscopy.

**Table 2:** Results obtained from the *P. falciparum* detection analysis on blood and serum paired samples from 26 patients (data are ordered by trophozoites). Three molecular methods—loop-mediated isothermal amplification (LAMP), rtPCR, and droplet digital PCR (ddPCR)—were used on both whole blood and serum. The parasitaemia (trophozoites/μL blood) was determined by microscopy.

Patient ID	Trophozoites/μL Blood	%iRBCs	LAMP Wholeblood	LAMP Serum	rtPCR (Ct) Wholeblood	rtPCR (Ct) Serum	ddPCR (Copies/μL Blood) Wholeblood	ddPCR (Copies/μL Serum) Serum
P1	383,460	9.15	Positive	Positive	20.05	34.85	9350	608.178
P2	366,000	9.5	Positive	Positive	21.89	21.42	5588	780.267
P3	196,930	4.7	Positive	Positive	21.69	37.12	5654	39.111
P4	159,544	2.96	Positive	Positive	18.88	24.58	21,109	107.36
P5	159,120	5.2	Positive	nv	20.37	nd	3916	221.467
P6	135,135	3.85	Positive	nv	19.07	33.95	8228	611.6
P7	118,800	3.37	Positive	Positive	16.89	23.66	82,500	337.92
P8	109,470	2.05	Positive	Positive	18.91	40.16	8668	nd
P9	97,600	2.44	Positive	Negative	18.51	nd	13,002	nd
P10	96,400	2.41	Positive	Positive	18.55	24.4	10,230	155.76
P11	32,560	0.55	Positive	nv	22.65	nd	2926	nd
P12	31,790	0.85	Positive	Negative	21.22	nd	8448	nd
P13	24,610	1.15	Positive	Negative	21.76	44.85	6644	0.152
P14	8076	0.184	Positive	Positive	23.51	nd	1476.2	268.4
P15	6409	0.15	Positive	Positive	28.42	32.93	80.52	80.178
P16	6133.5	0.1557	Positive	Positive	21.15	25.08	7601	63.184
P17	4620	0.092	Positive	Positive	26.89	30.86	191.4	2.249
P18	2764	0.0507	Positive	Positive	26.31	27.14	242	108.044
P19	2613	0.0549	Positive	nv	26.04	nd	391.6	7.676
P20	332.5	0.0088	Positive	Positive	27.29	29.8	25.74	5.104
P21	189	0.0043	Positive	Positive	30.83	45.26	14.3	0.093
P22	95.4	0.0023	Positive	Negative	31.45	nd	11.88	0.152
P23	76.5	0.0014	Positive	Negative	33.38	nd	1.54	nd
P24	39.5	0.0008	Positive	Positive	31.76	40.29	9.24	1.662
P25	17.5	0.0003	Positive	Negative	34.5	nd	1.276	nd
P26	17	0.0004	Positive	Positive	30.7	32.22	5.72	0.484

Foot note: parasitaemia is expressed as the number of trophozoites/μL of blood and the % of iRBCs as counted with microscopy. iRBCs, infected erythrocytes; nd, not detected; nv, non-valid.

## Supplementary Materials

The following are available online at https://www.mdpi.com/2076-0817/9/6/478/s1, Figure S1: Quantitation by real-time PCR (rtPCR) (**A**) and ddPCR (**B**) of a *P. falciparum* sample diluted in whole blood before DNA extraction. Parasitaemia data was reported as Log(number of parasites/μL), rtPCR and ddPCR data was reported as Ct value and Log(copies/μL respectively). Figure S2: Results of the statistical model to predict microscopy counts from ddPCR copies/μL (P27–P33) from our dataset and kP1-kP28 from [26]. Solid lines represents 95% confidence intervals, blue filled dots predicted values and red filled squares observed data. Table S1: Full clinical and demographic descriptions of 33 malaria patients (ordered by parasitaemia), Table S2: real-time PCR (rtPCR) and ddPCR data of P. falciparum DNA detection in infected blood using serial dilution., Table S3: Predictive analysis using microscopy counting of parasites/µL of blood and ddPCR quantitation of gene copies/µL of blood., Table S4: Primers and probe used in rtPCR and ddPCR. All the data generated or analyzed during this study are included in this published article (and its supplementary information files).
