# Supplementary material for: Droplet Digital PCR for the Detection of Plasmodium falciparum DNA in Whole Blood and Serum: A Comparative Analysis with Other Molecular Methods
Source: Pathogens. 2020 Jun 17;9(6):478. doi: 10.3390/pathogens9060478 (PMC7350319; doi:10.3390/pathogens9060478)
Supplement: Supplementary file 1 [file pathogens-09-00478-s001.zip › Suppl material proof/Table S4.docx]

**Table S4.** Primers and probe used in rtPCR and ddPCR.

| **Organism** | **Primer/Probe** | **Sequence** | **Ref** |
| --- | --- | --- | --- |
| *Plasmodium falciparum* | FAL-F | 5′-CTTTTGAGAGGTTTTGTTACTTTGAGTAA-3’ | [33] |
|  | FAL-R | 5′-TATTCCATGCTGTAGTATTCAAACACAA-3’ |  |
|  | FAL-probe | FAM-5’-TGTTCATAACAGACGGGTAGTCATGATTGAGTTCA-3’-MGB |  |
| *Homo sapiens* | *β actin*-F | 5’-ACCGAGCGCGGCTACAG-3’ | [34] |
|  | *β actin*-R | 5’-CTTAATGTCACGCACGATTTCC-3’ |  |
|  | *β actin*-probe | HEX-5’-TTCACCACCACGGCCGAGC-3’-TAMRA |  |
