# Supplementary figures and images for: Droplet Digital PCR for the Detection of Plasmodium falciparum DNA in Whole Blood and Serum: A Comparative Analysis with Other Molecular Methods
Source: Pathogens. 2020 Jun 17;9(6):478. doi: 10.3390/pathogens9060478 (PMC7350319; doi:10.3390/pathogens9060478)

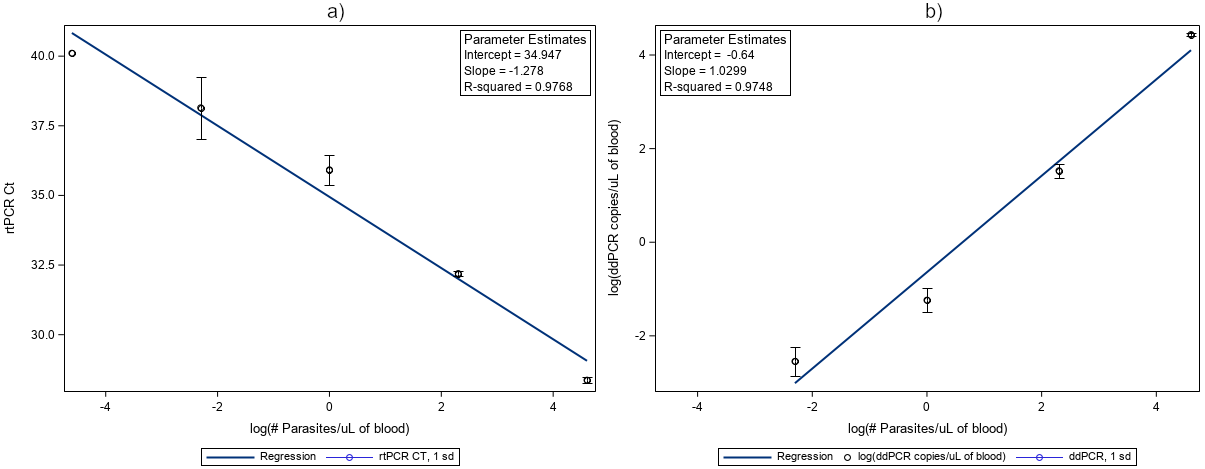

Supplement: Supplementary file 1 [file pathogens-09-00478-s001.zip › Suppl material proof/Figure S1.tif]

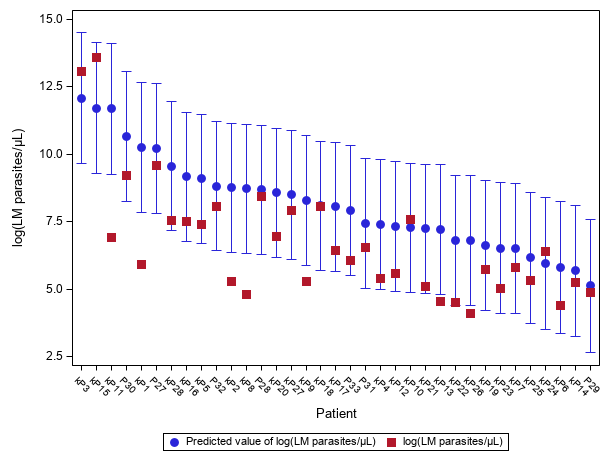

Supplement: Supplementary file 1 [file pathogens-09-00478-s001.zip › Suppl material proof/Figure S2.tiff]
